# Epigallocatechin-3-Gallate and Genistein for Decreasing Gut Dysbiosis, Inhibiting Inflammasomes, and Aiding Autophagy in Alzheimer’s Disease

**DOI:** 10.3390/brainsci14010096

**Published:** 2024-01-19

**Authors:** Ahalya Muraleedharan, Swapan K. Ray

**Affiliations:** 1 Department of Chemistry and Biochemistry, University of South Carolina, Columbia, SC 29208, USA; ahalya@email.sc.edu; 2 Department of Pathology, Microbiology, and Immunology, University of South Carolina School of Medicine, Columbia, SC 29209, USA

**Keywords:** Alzheimer’s disease (AD), autophagy, bioflavonoids, epigallocatechin-3-gallate (EGCG), genistein (GS), gut microbiome, gut dysbiosis, inflammasomes

## Abstract

There are approximately 24 million cases of Alzheimer’s disease (AD) worldwide, and the number of cases is expected to increase four-fold by 2050. AD is a neurodegenerative disease that leads to severe dementia in most patients. There are several neuropathological signs of AD, such as deposition of amyloid beta (Aβ) plaques, formation of neurofibrillary tangles (NFTs), neuronal loss, activation of inflammasomes, and declining autophagy. Several of these hallmarks are linked to the gut microbiome. The gastrointestinal (GI) tract contains microbial diversity, which is important in regulating several functions in the brain via the gut-brain axis (GBA). The disruption of the balance in the gut microbiota is known as gut dysbiosis. Recent studies strongly support that targeting gut dysbiosis with selective bioflavonoids is a highly plausible solution to attenuate activation of inflammasomes (contributing to neuroinflammation) and resume autophagy (a cellular mechanism for lysosomal degradation of the damaged components and recycling of building blocks) to stop AD pathogenesis. This review is focused on two bioflavonoids, specifically epigallocatechin-3-gallate (EGCG) and genistein (GS), as a possible new paradigm of treatment for maintaining healthy gut microbiota in AD due to their implications in modulating crucial AD signaling pathways. The combination of EGCG and GS has a higher potential than either agent alone to attenuate the signaling pathways implicated in AD pathogenesis. The effects of EGCG and GS on altering gut microbiota and GBA were also explored, along with conclusions from various delivery methods to increase the bioavailability of these bioflavonoids in the body.

## 1. Introduction

Alzheimer’s Disease (AD), the most common type of dementia, is known as a neurodegenerative disease caused by the accumulation of amyloid beta (Aβ) peptides and tau protein hyperphosphorylation resulting in the formation of neurofibrillary tangles [1,2]. AD is the seventh leading cause of death in the United States (US) [3]. Currently, about 6 million Americans have AD, mostly affecting people above the age of 65 [3]. With increasing age, the likelihood of occurrence of AD also increases, with 32% of people above the age of 84 years being diagnosed with AD [4]. This review article is focused on AD specifically because it is the most common neurodegenerative disease. Besides, other neurodegenerative diseases have similar pathogenesis in terms of protein accumulation and inflammation; hence, a new therapeutic strategy targeted to AD may also be applied to treat similar conditions. There are two existing categories of biomarkers that are used to identify AD in a patient. The first one is a biomarker detected in the brain amyloid using cerebrospinal fluid (CSF) and positron emission tomography (PET) imaging measurements [1]. The second category involves spotting in CSF the biomarker tau that relates to neuronal injury, using fluorodeoxyglucose (FDG) to analyze metabolic activity, and performing magnetic resonance imaging (MRI) to measure brain atrophy [1].

Apart from dementia, many patients also have non-amnestic pathogenesis involving dysfunction in visual, language, and behavioral domains [2]. The phases of AD can be split into multiple stages. First, the pre-symptomatic stage (a few years in length), in which the patient only has mild amnesia and has no signs of AD, but detecting even a single marker of brain amyloidosis in CSF and PET is enough to be diagnosed with AD [5,6,7]. At the beginning of the disease progression, Aβ plaques are formed in the basal, temporal, and orbitofrontal neocortex regions of the brain, while Aβ plaques triggered tau tangle formation takes place in locus coeruleus and trans entorhinal and entorhinal areas [8]. Second, mild stage during which the patients develop amnesia enough to have impediments in their daily lives. Third, moderate stage, in which amnesia worsens to the point of dysfunction in recognizing friends and family. Fourth, a severe stage during which the patient can lose functional abilities, becoming bedridden and resulting in death [5]. During critical stages, Aβ plaques are hypothesized to spread to the mesencephalon, lower brain stem, and cerebellar cortex, while the neurofibrillary tangles (NFTs) spread to the hippocampus and neocortex regions of the brain [8]. Apart from Aβ accumulation, other factors such as tau aggregation, neuroinflammation, and oxidative stress can lead to neurodegeneration in AD. No treatment can completely cure AD. However, there are temporary treatments (prescription drugs) such as cholinesterase inhibitors (Donepezil, Rivastigmine, and Galantamine), glutamate regulators (Memantine), and a combination of a cholinesterase inhibitor and a glutamate regulator (Donepezil and memantine) that alleviate the AD symptoms [9].

The pathogenesis in AD is associated with an alteration in gut microbiota or gut dysbiosis that affects brain functions via the gut-brain-axis (GBA), resulting in the activation of inflammasomes that cause neuroinflammation, and diminishing autophagy that fails to clean up and recycle damaged and non-functional cellular components, all of which contribute to neurodegeneration and cognitive decline [8]. In the present time, available synthetic drugs and other therapeutic agents temporarily alleviate AD symptoms rather than eliminate the root causes of AD. Besides, AD therapeutic agents on the market are often associated with severe side effects, limiting their long-term use for the treatment of AD. Recent findings indicate that bioflavonoids, especially epigallocatechin-3-gallate (EGCG) and genistein (GS), are highly promising for targeting gut dysbiosis, inhibiting neuroinflammation, and promoting the cellular recycling mechanism autophagy leading to neuroprotection and prevention of pathogenesis in AD. This review article will focus on the meta-analysis of recent advances in the use of ECCG and GS in controlling AD pathogenesis in vitro and in vivo, leading to a rationale for future exploration of the efficacy of these bioflavonoids in AD in clinical settings.

## 2. Prescription Therapeutic Options for AD

The cholinergic hypothesis states that the onset of AD progresses due to the decrease in acetylcholine (ACh) synthesis [10]. Hence, this therapeutic strategy intends to inhibit the activity of acetylcholinesterase enzyme (AChE), which otherwise degrades ACh, to increase the cholinergic signaling in the brain. By deterring the degradation of ACh at the synapses, the cholinergic receptors stay activated [5]. To inhibit the AChE, varying AChE inhibitors have been created, such as Physostigmine, Tacrine, Donepezil, Rivastigmine, Galantamine, and Metrifonate. Among these inhibitors, only four drugs as potential therapeutics, including Donepezil (AChE inhibitor), Galantamine (AChE inhibitor), Rivastigmine (reverse inhibitor of both AChE and butyrylcholinesterase or BChE), and Memantine are currently available in the market for use in the AD patients (Table 1). However, all these drugs have side effects, which increase with the increasing dosage administered [10].

One of the AChE inhibitors, Physostigmine, was recalled from being used as an AD therapeutic drug due to the severe side effects it caused. With a dosage of 36 mg/d, more than 50% of AD patients were nauseous and vomiting, while 20–50% of patients were having diarrhea and dizziness [11]. Tacrine, an inhibitor of both AChE and BChE, was the US Food and Drug Administration (FDA)-approved drug until 2013, and then it was discontinued due to the side effects of high doses administered [10]. Around 20–50% of AD patients who were given Tacrine (dosage of 80–160 mg/d) experienced nausea and, most importantly, developed abnormal liver functionality, and 10–20% of the patients endured vomiting, diarrhea, dizziness, and anorexia [10]. The next inhibitor, Donepezil, is still prevalently used in AD patients, ranging from moderate to severe cases [12]. In the case of Donepezil, the side effects presented are heavily dosage-based. According to a double-blind patient study conducted, patients who were administered the 10 mg/d Donepezil showcased more cognitive improvements compared to patients who received the 5 mg/d dosage [12]. Donepezil helps control the early activation of inflammatory cytokines along with a reduction in oxidative stress [10]. Compared to Tacrine, Donepezil also has a longer half-life, resulting in a decrease in cholinergic side effects [12]. Another inhibitor that is also used in mild AD cases is Rivastigmine. When a dosage of 6–12 mg/d is administered, more than 50% of AD patients feel nauseous, and 20–50% of patients experience vomiting and diarrhea [11]. The side effects in case of overdose are irregular heartbeat and chest pain [10]. Derived from the daffodil bulbs, Galantamine was used for central nervous system (CNS) defects for years before becoming an FDA-approved drug for the treatment of AD in 2001. The mechanism of Galantamine involves binding to nicotinic cholinergic receptors to inhibit AChE. Galantamine causes fewer side effects when compared to Physostigmine, Tacrine, Donepezil, and Rivastigmine. At a dosage of 20–50 mg/d, 10–20% of AD patients experience nausea and vomiting, while only 10% of patients have diarrhea and dizziness [11]. As an organophosphate AChE inhibitor, Metrifonate is efficient in cognitive improvements, but it is associated with severe side effects such as bradycardia, rhinitis, and abdominal pain. In rare cases, neuromuscular dysfunction and respiratory failure have also occurred, leading to Metrifonate being withdrawn from being used in AD treatment [13].

## 3. Bioflavonoids as Novel Therapeutic Option for AD

As mentioned above, there are varying potential categories for therapeutic values of AChE inhibitors in the treatment of AD. However, natural compounds derived from plants are currently gaining wide attention for the treatment of AD and other neurodegenerative diseases in vitro and in vivo. Polyphenols are known to have enormous potential to regulate diversity as well as the composition of gut microbiota, which is associated with neurological health. Studies have showcased that polyphenols can reduce the neurological deficits that are caused due to neuroinflammation [14,15,16]. Bioflavonoids are a group of natural polyphenolic compounds that are derived from fruits and vegetables. A few examples of fruits and vegetables would include apples, onions, mulberries, and bilberries. Bioflavonoids are popularly consumed via tea, beer, and wine [17]. This subclass of polyphenols of biological origin is implicated in anti-apoptotic and pro-survival signaling pathways and decreasing the pathological effects of AD [18,19]. Among the 5000 types of flavonoids, which are mostly found in plants, there are six main types of flavonoids such as flavonols, flavones, flavan-3-ols, flavanones, anthocyanidins, and isoflavones [20,21]. Bioflavonoids, which are exclusively derived from biological origins (mainly plants), are also well known for showcasing anti-inflammatory, anti-viral, anti-apoptotic, anti-platelet, and anti-tumoral properties [17,22,23].

**Figure 1:**
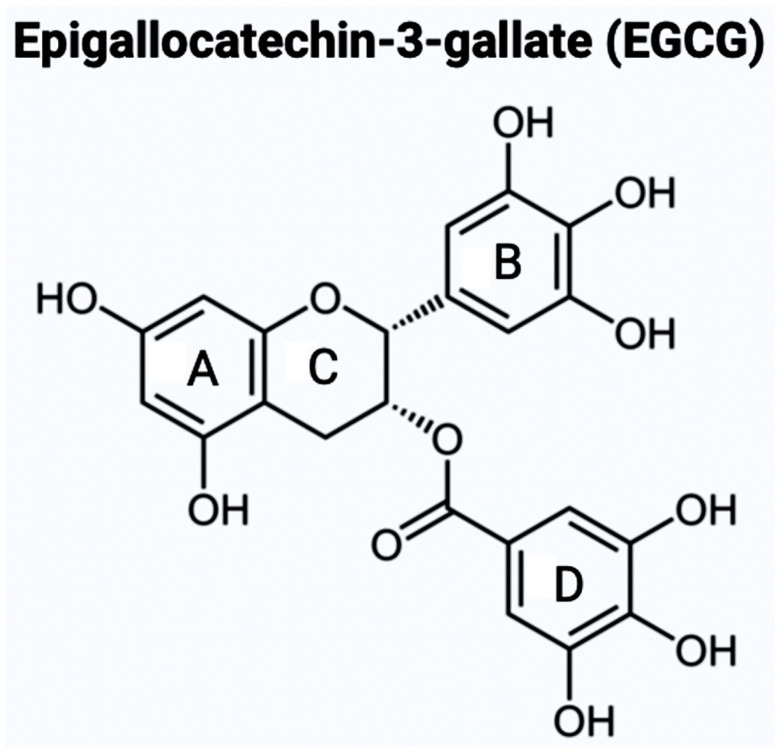
It illustrates the chemical structure of epigallocatechin-3-gallate (EGCG) with the four rings (A, B, C, and D). The B ring feature increases the probability of EGCG being used as an AChE inhibitor. This chemical structure was created with BioRender.com.

Catechins are a group of bioflavonoids that can be extracted from tea, and this group includes epigallocatechin (EGC), epicatechin gallate (ECG), epicatechin (EC), and the most abundant compound EGCG [23]. As shown in Figure 1, the chemical structure of EGCG contains A, B, C, and D rings produced from the esterification of EGC with gallic acid [24]. Both the A and C rings have a phenyl group at C2 and a gallate group at C3 positions. The B and D rings of EGCG contain 3,4,5-trihydroxy groups, which have the potential for proteasome activity in vitro [24]. On the aromatic B ring, catechins have di- or tri-hydroxyl groups along with meta-5,7-dihydroxyl groups on the A ring [25]. The presence of phenolic groups in these compounds increases their antioxidant properties. The structure of flavonoids is important in creating a novel therapeutic drug for the treatment of AD.

**Figure 2:**
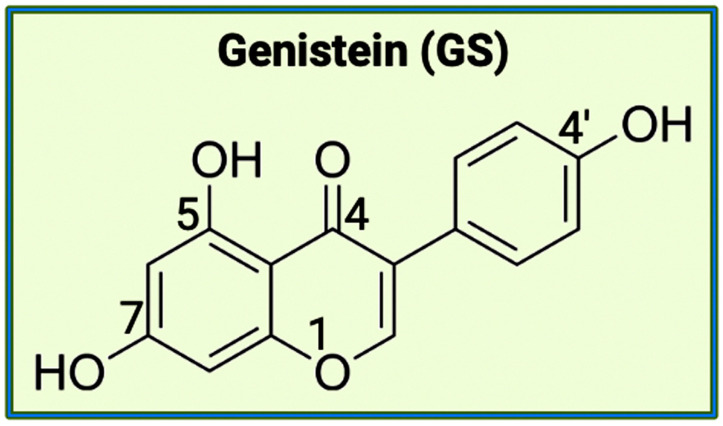
The chemical structure of genistein (4′,5,7-trihydroxy-isoflavone). This isoflavone, mostly present in soybeans, has the potential to behave as a weak estrogen or as an anti-estrogen, along with being implicated in varying biological activities (e.g., apoptosis and cell differentiation). This chemical structure was created with BioRender.com.

The aromatic B ring containing -OH groups mimics the structure of Donepezil, allowing the compound to bind with the peripheral anionic site (PAS) of the AChE gorge [25]. Apart from EGCG, the second bioflavonoid that we will be examining for the treatment of AD is GS. While EGCG is part of the catechins subgroup, GS belongs to the subgroup called isoflavones [26]. GS has a structure like estrogen and has the capacity to function as an anti-estrogen. This plant-derived compound has an extensive history of anti-inflammatory properties (Figure 2). A crucial component of GS’s endocrine effects is due to its similarity to S-equol, a phytoestrogen produced in the intestinal microbiota [27].

Further studies conducted showcase the inhibitory functions of GS with an important cell signaling pathway involving the nuclear factor kappa-B (NF-κB), which is crucial for promoting inflammation. GS has also been implicated in other cell signaling pathways involving prostaglandins (PGs), inducible nitric oxide synthase (iNOS), pro-inflammatory cytokines, and reactive oxygen species (ROS) [26]. Based on the exhibited properties of EGCG and GS, both bioflavonoids should be explored further regarding their potential to become novel therapeutic drugs for the treatment of neurodegenerative diseases, including AD. EGCG and GS can be used as readily available alternatives for AChE inhibition because they are natural substances derived from plants. However, EGCG and GS can also be useful to target the gut microbiome through varying delivery methods. A function attributed to polyphenols is their ability to foster the growth of beneficial bacteria (*Lactobacillus* and *Bifidobacteria*) in the gut microbiome while limiting the pathogenic bacteria (*Bacteroides* and *Clostridia*) [28].

As stated earlier, there are existing synthetic therapeutic drugs to palliate the pathogenic symptoms of AD, especially for their function as inhibitors of AChE to increase the production of ACh levels. However, as also stated above, there are notable side effects associated with these AChE inhibitors, making them untenable for long-term use, which is why the most prominent bioflavonoids, EGCG and GS, seem to pledge to provide a new paradigm as suitable as AChE inhibitors. Apart from targeting AChE, EGCG and GS are also highly regarded for promoting the favorable condition of the gut microbiota and, thus, decreasing gut dysbiosis (an imbalance in types of natural microflora in the gut) that otherwise causes neuropathogenesis. The focus of this review article is to explore EGCG and GS as the potential treatments through varying methods (AChE inhibitor, diet, fecal microbiota transplantation, neural stem cell therapy, and nanomaterials) to target the dysbiosis in the gut microbiome, which has a considerable influence on the course of AD.

## 4. An Overview of the Gut Microbiota

The gut microbiota in every individual is unique, and thus, it is determined through environmental factors rather than being a genetically inheritable trait. In the gut microbiota, the staggering microbial diversity and colonization result in varying complex interactions, diseases, and immune responses. Depending on the area of the gastrointestinal tract being examined, the density and diversity of the gut bacteria fluctuate due to the difference in the local conditions [29]. A proper understanding of the function of microbiota in the gut is crucial for the development of successful therapeutics to target neurological disorders.

There are diverse groups of gut members (microbiota, microbial structural elements, microbial metabolites, and internal/external structural elements) that morph into the concept of the microbiome [28]. Gut microbiota contributes to all the living organisms present in the microbiome, such as bacteria, archaea, fungi, protists, and algae. In comparison, microbial structural elements include a variety of components such as proteins, lipids, polysaccharides, nucleic acids, and other genetic elements. Signaling molecules, toxins, and organic molecules generate a group of microbial metabolites. Additionally, environmental conditions also affect the ecological niche of the human body, resulting in the variability of the microbiome in people [28]. In the human body, microbial cells are as abundantly found as somatic cells. At the current estimation, there are 500–1000 or more species of bacteria existing in the human body [28]. In gut microbiota, the existing bacteria belong to two different phyla called *Bacteroidetes* and *Firmicutes*, and both phyla consist of more than 200 genera [29,30]. The extent of microbial colonization for versatile roles in the human body can be appreciated through an approximate estimate of 2,000,000 bacterial genes in the gut compared to about 20,000 human genes [28]. However, due to the scale and complexity of the microbial diversity present, the factors and influences affecting the conditions of the microbiome, along with the resulting interactions with the immune, endocrine, and nervous systems, are being heavily researched in many disease pathologies including AD.

### 4.1. Activity of Gut Microbiota in the Human Body

The intestinal bacteria produce short-chain fatty acids (SCFAs) through the fermentation of non-digestible carbohydrates (NDC) and dietary fiber, and these SCFAs are formate, acetate, propionate, and butyrate, with the presence of acetate being three times higher [30,31,32]. Specifically, an increased presence of formate has been linked to the possibility of higher inflammation [32]. In a study of the introduction of butyrate to isolated germ-free colonocytes, the rate of oxidative phosphorylation increased while autophagy decreased [31]. For fermentation, the source of the carbohydrates used is the ones that were not digested or absorbed in the small intestine [32]. The functions of SCFAs include affecting cellular processes such as gene expression, differentiation, proliferation, and apoptosis [31]. The SCFAs, which are produced from NDC and dietary fiber, also regulate the permeability of the gut and blood-brain barriers [33].

In the CNS, SCFAs play a key role during the production of neural progenitor cells (NPCs) that produce neuronal and glial cell types [34,35]. According to a study conducted recently, the increased concentration of SCFAs positively affected the expression of genes involved in the proliferation of NPCs [36]. Free fatty acids (FFAs), which are the products of the metabolic pathways, are known to function as signaling molecules via interaction with free fatty acid receptors (FFARs) that form a family of G protein-coupled receptors (GPCRs). As the largest group of transmembrane proteins, GPCRs are currently known to be the most successful drug targets. There are a few mechanisms that are regulated by SCFAs to increase the production of NPCs. The first mechanism involves specific FFARs (i.e., GPCRs), most notably FFAR2 (GPR43) and FFAR3 (GPR41), being upregulated due to increased exposure to SCFAs. In the second mechanism, SCFAs regulate the physiological pH that modulates neurodevelopmental effects along with anti-apoptotic effects [36]. This showcases the importance of further exploring the influence of the SCFAs in the gut microbiome regarding diseases, especially neurologically related.

SCFAs are produced in the intestinal tract due to the metabolic activities of the diverse and thriving bacteria population there. The concentrations of SCFAs vary based on the location in the gut [31]. In the proximal colon, 70 to 140 mM SCFAs are present, and in the distal colon, they are about 20 to 70 mM with increased production of acetate. Additionally, SCFAs have established themselves in the oral cavity as well, with varying concentrations of the different fatty acids (6 to 38 mM acetate, 1 to 13 mM propionate, and 0 to 5 mM butyrate). In the lower female genital tract, the acetate concentration can reach up to 120 mM, which is directly influenced by infection or inflammation [31].

Regarding inflammation, SCFAs modulate the production of cytokines, which are crucial to control inflammation [30]. Besides cytokines, SCFAs also regulate other immune cells, including macrophages, neutrophils, and dendritic cells. According to studies, *Faecalibacterium prausnitzii* (also called *F. prausnitzii*, a strictly anaerobic bacterium) has an inflammatory protein that can inhibit NF-κB family of transcription factors (present in the intestinal epithelial cells of the animals). This transcription factor family begins the transcription of target genes by binding to a specific DNA element, κB enhancer. After one of the five transcription factors belonging to the family activates the κB enhancer, the phosphorylated inhibitor of NF-κB or IκB is degraded using a proteasome. This results in the NF-κB being freed from the cytoplasm and moved to the nucleus to activate pro-inflammatory genes to heal tissue damage through cytokine production [30,37]. The importance of NF-κB pathway in targeting pro-inflammatory genes can lead to an overactivation of this pathway, resulting in higher levels of inflammation [38]. To suppress the overproduction of inflammatory cytokines, the gut microbiota can inhibit the NF-κB pathway using the microbe-derived factors [38].

### 4.2. Onset Factors in the Microbiota for Dysbiosis

Dysbiosis occurs when the normal state of the gut microbiota is unbalanced due to varying factors. One of the main examples is when the anti-inflammatory cytokines and the pro-inflammatory cytokines produced by the microbes are not balanced, then dysbiosis takes place. This imbalance can result in conditions including inflammatory bowel disease (IBD), irritable bowel syndrome (IBS), diabetes, obesity, cancer, cardiovascular problems, and many CNS disorders [39]. Both IBD and IBS are incredibly challenging conditions. There are three types of dysbiosis: type 1 indicates a decrease of beneficial bacteria, type 2 shows an increase of pathogenic bacteria, and type 3 states a decrease in overall bacterial diversity [40].

The number of factors that can directly affect dysbiosis are many such as diet, birthing conditions (e.g., mode of birth, antibiotic exposure, and hygiene), chemical exposure, psychological and environmental stimuli (e.g., pathogens, sleep deprivation, circadian rhythm dysfunction, toxins, and noise), temperature, and intestinal infection. Diet is one of the crucial regulators of the gut microbiota [30]. In studies comparing a ‘Western diet’ (high animal protein, high in sugar and saturated fats) and an ‘agrarian diet’ (low animal protein, low levels of saturated fat and simple sugars), the results displayed the ‘Western diet’ leading to dysbiosis and lower levels of SCFAs [41]. On the other hand, the ‘agrarian diet’ results in more production of SCFAs and higher gut bacteria diversity, which helps limit the growth of potentially pathogenic bacteria that otherwise lead to diseases such as IBD [41]. The reason why the lower animal protein levels in an ‘agrarian diet’ help with the gut microbiota is due to the side effects of protein and amino acid fermentation [42]. When more protein is consumed, the gut must shift to increase the pH to break down the proteins that result in the production of compounds, including hydrogen sulfide, reactive oxygen species, and ammonia, which are unhealthy for the gut [42,43]. An increased intake of Vitamin D can help inhibit inflammatory responses along with modulating the state of the gut microbiota. When mice lack Vitamin D, the intestinal epithelial barrier is not protected, resulting in the growth of pathogenic bacteria in the gut and the initiation of inflammation due to dysbiosis [41].

Apart from the influence of diet on dysbiosis, environmental factors are also implicated in this process, taking place in the microbiota. Beginning from birth, a person’s gut flora is affected based on whether they were born vaginally or via a c-section [44]. Studies have showcased a relationship between how likely a child is to develop obesity/diabetes and whether they were born vaginally [45]. In the case of children who were born vaginally, they were reported to have been exposed to the mother’s beneficial bacteria present in the birth canal and rectum. On the other hand, c-section babies were stated to be at a higher risk of developing diabetes because they were only exposed to pathogenic bacteria [45]. The factors that cause dysbiosis in the gut microbiota are diverse, so exploring the gut flora in relation to diseases is crucial.

### 4.3. Gut-Brain Axis (GBA) and Dysbiosis

**Figure 3:**
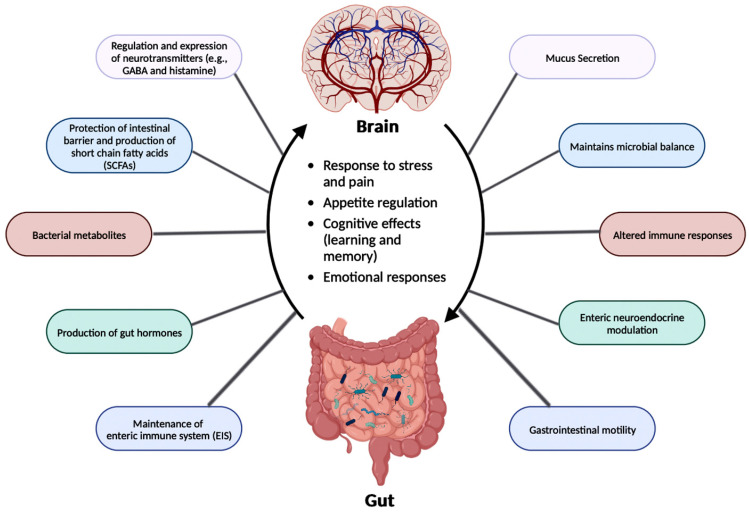
The gut-brain axis (GBA) participates in the regulation of various functions. The link between the brain and the gut flora is important for the regulation of essential functions in the human body, such as the production of neurotransmitters, SCFAs, bacterial metabolites, and gut hormones. GBA is also essential in maintaining microbial balance along with the enteric immune system (EIS). This figure was created with BioRender.com.

The relationship between the gut microbiota and the brain is called the Gut-Brain Axis (GBA), and this two-way communication is built using immune, circulatory, and neural pathways (Figure 3) [34]. GBA connects the CNS (comprised of brain and spinal cord), autonomic nervous system (ANS), enteric nervous system (ENS), and hypothalamic pituitary adrenal (HPA) axis [46]. Particularly, the function of the HPA axis includes regulating the adaptive responses from the body to any stressors needed [46,47]. An increase in the occurrence of inflammatory cytokines such as interleukin-1 beta (IL-1β), IL-6, and tumor necrosis factor alpha (TNF-α) through the production of corticotropin-releasing factor (CRF) and adrenocorticotropic hormone (ACTH) is an example of environmental stress that can activate the HPA axis. The bidirectional communication line results in the regulation of intestinal functional effector cells (immune cells, epithelial cells, enteric neurons, smooth muscle cells, interstitial cells of Cajal, and enterochromaffin cells) [46]. Unsurprisingly, the gut microbiota has been implicated in affecting the bidirectional communication between the gut and the brain [46]. The microbes present in the gut produce metabolites such as SCFAs, gamma-aminobutyric acid (GABA), tryptophan, serotonin, catecholamines, metabolites of bile acids and neurotransmitters, and cytokines that can signal to the receptors present in the gut [48]. The sequence of the events leading up to dysbiosis in the gut has not yet been properly established and can be caused by varying stresses. The dysbiosis of the gut microflora causes an increase in the gut and blood-brain barrier permeability, production of bacterial amyloids, and formation of lipopolysaccharides (LPS) leading up to the deposition of amyloid fibrils in the brain, resulting in the pathogenesis (neuroinflammation, cognitive decline) of neurological disorders such as AD and stroke [49]. There still has not been a complete understanding of the pathways involved in the GBA bidirectional communication line.

However, there are a few pathways that are highly conserved for certain functions. One of the physical pathways connecting the gut and the brain is the vagus nerve (tenth cranial nerve) [50]. The vagus nerve is colloquially known as the communication superhighway in the body. Having the efferent and afferent neurons present in this nerve, it transports the motor signals traveling between the brain and other body organs. An example of an organ would be the gut microbiome, allowing the brain to detect any imbalance in the gut flora [50]. In the CNS, the dysregulation of GABA, an inhibitory neurotransmitter, is known to cause mental disorders such as schizophrenia, autism, and depression [34]. The gut microbiota controls the regulation of GABA signaling through the vagus nerve. Based on a clinical study conducted, only in mice with a vagus nerve present did the expression of GABA increase, leading to a decrease in depressive symptoms [34]. Another critical aspect of the gut microbiome is its influence on both innate and adaptive immunity and, thus, inflammation. As stated above, the gut microbiota’s ability to regulate the HPA axis opens the possibility of disorders such as anxiety and depression manifesting the involvement of the gut hormones, which control a person’s mood. Apart from gut hormones, neurotransmitters (e.g., dopamine, serotonin, noradrenaline, and GABA) may also function as hormones in situations and are regulated by the gut microbiota even though these neurotransmitters are not exclusively produced in the gut [50]. The varying functions of the microbiota regarding the immune system and the hormonal changes, including the consequences of a dysfunction in the gut flora, display the importance of analyzing the relationship of the gut microbiota in the context of neurological diseases such as AD.

### 4.4. GBA and AD

The dysbiosis in gut microbiota directly affects the GBA, which is linked to AD clinical symptoms such as Aβ plaque deposition, cognitive decline, and memory loss (Table 2). To explore the change in the microbiota and the Aβ plaque deposition in the brain, studies created AD animal models comparing the microorganisms and SCFAs in fecal samples between AD mice and wild-type animals [51]. The AD mice displayed lower levels of SCFAs, which had the potential to alter multiple metabolic pathways along with increasing the deposition of Aβ plaques [51]. Another study for analyzing the effect of age compared the double transgenic (TG) mice expressing a chimeric mouse/human amyloid precursor protein (APP) and a mutant human presenilin 1 (PSEN1), both APP/PSEN1 mutations ensured an early-onset of D, and C57BL/6 wild-type (WT) mice, and the results unveiled the potential of targeting the gut microbiota in AD animals [52]. The 6-month-old APP/PSEN1 mice, with their gut microflora documented differently from the WT mice, experienced cognitive decline [52]. The microbial diversity of the APP/PSEN1 mice deteriorated along with age with increases in the population of bacteria from the *Helicobacteraceae* and *Desulfovibrionaceae* families [52]. In the *Helicobacteraceae* family, *Helicobacter pylori* (*H. pylori*) participates in causing dysbiosis resulting in gastric disorders such as chronic active gastritis, peptic ulcer disease (PUD), mucosa-associated lymphoid tissue (MALT) lymphoma and gastric carcinoma [53]. Interestingly, a recent study revealed inserting *Desulfovibrio* stains in *Caenorhabditis elegans* (*C. elegans*) increased the number of alpha-synuclein aggregates causing Parkinson’s disease (PD), another prominent neurodegenerative disease [54]. The decrease in microbial diversity in the APP/PSEN1 mice highlights the importance of regulating the gut microbiome as a viable therapeutic target for the treatment of AD.

Apart from the animal models, the resultant consequence of dysbiosis causing AD can be confirmed in patient studies. Again, fecal matter was examined in AD patients and AD-lacking participants [52]. When comparing the fecal samples from AD patients and AD-lacking participants, the microbial diversity exhibited by the AD patients is comparatively lower. Patients with AD had fewer *Firmicutes* and *Bifidobacterium* and excessive amounts of *Bacteroidetes*. Dysfunction in the production and metabolism of bile acid (BA) causes cognitive decline. Studies also show AD patients have a higher amount of secondary BA compared to lower levels of primary BA. Secondary BA, which results from the removal of 7α-hydroxy or 7β-hydroxy group from primary BA, causes toxicity to specific *Lactobacillus* species. The gut microbiota activates 7α-dehydroxylation of cholic acid, resulting in the upregulation of deoxycholic acid and altered conformations of glycine and taurine [52]. Further findings also strengthened the association of amyloid plaque accumulation with pro-inflammatory gut bacteria. A study highlighted the abundance of pro-inflammatory gut bacteria (*Escherichia* and *Shigella*) and a decrease in anti-inflammatory gut bacteria (*E. rectale*) in the fecal matter of elderly patients with cognitive dysfunction [58]. The gut microbiota is extensively involved in crucial aspects of cognitive deterioration in AD due to an increase in neuroinflammation and a decrease in autophagy.

Beyond hypothesizing the link between the gut microbiota and the brain regarding AD pathogenesis, there are recent studies that display a possibility of AD development beginning in the gut before reaching the brain. In the gastric wall of mice, Aβ_1–42_ oligomers were administered to induce amyloidosis [59]. The amyloid was observed using immunofluorescence staining and in vivo imaging to have traveled from the gut to the brain [59]. Various AD hallmarks were observed in the mice, such as cerebral and vagal beta-amyloidosis. Another remarkable finding was gastric dysfunction, which was usually attributed to PD symptoms [59]. Consequently, the exploration of the origin of AD pathogenesis should be extended beyond focusing on the brain and include the gut microbiota.

## 5. Neuroinflammation in AD

Most neurological conditions, such as autism spectrum disorders (ASD), epilepsy, PD, cerebrovascular diseases, and AD, have aspects of neuroinflammation as part of their pathogenesis. Cytokines, produced by microglia and astrocytes, are the central factors that influence all characteristics of neuroinflammation, ranging from pro-inflammatory and anti-inflammatory processes to neuronal injury [60].

In the case of neurological disorders, microglial activation results in the production of pro-inflammatory cytokines (e.g., IL-1, IL-6, and TNF-α), and overproduction of these molecules is toxic to neurons and glial cells [61]. In AD, Aβ forms soluble oligomers and fibrils, which bind to microglia through cell-surface receptors, including the scavenger receptor class a1 (SCARA1), a cluster of differentiation 36 (CD36), CD14, a6β1 integrin, CD47, and Toll-like receptors (TLRs). Hence, the deletion of CD36, TLR4, or TLR6 can decrease Aβ-induced cytokine production [61]. Another source of cytokines is astrocytes, which regulate synaptic transmission through reactive astrogliosis [61]. The activation of astrocytes can happen because of multiple triggers, such as transforming growth factor-beta 1 (TGF-β1), leukemia inhibitory factor (LIF), and ciliary neurotrophic factor [62]. After activation, astrocytes either become neurotoxic A1 producing the pro-inflammatory molecules such as reactive oxygen species (ROS), IL-6, IL-1β, and TNF-α or become neuroprotective A2 along with the protective factors such as vascular endothelial growth factor (VEGF), brain-derived neurotrophic factor (BDNF), and nerve growth factor (NGF) [62]. The exposure of astrocytes to initial Aβ deposits upregulates the expression of Aβ proteases such as neprilysin, insulin-degrading enzyme (IDE), endothelin-converting enzymes (ECE), and angiotensin-converting enzyme (ACE) [60]. In studies exploring animal models of AD, astroglia atrophy was detected in the initial stages. This atrophy, along with dysfunction of Aβ proteases, can cause a decrease in proteolytic clearance of Aβ. Uncontrolled cytokine production negatively impacts the long-term potentiation (LTP) of synaptic transmission, aiding the symptomatic progression of neurodegenerative diseases and increasing intestinal permeability [60,63]. The importance of controlling inflammation in AD could be understood through the example of a clinical study in which AD patients were administered non-steroidal anti-inflammatory drugs (NSAIDs) in the Baltimore longitudinal study, and these patients experienced less cognitive decline compared to the patients who were administered aspirin [64]. Due to the impact of pro-inflammation cytokines produced by both microglia and astrocytes, neuroinflammation is an important target when creating a potential therapeutic drug for AD.

### 5.1. Implications of Gut Microbiota in Neuroinflammation in AD

**Figure 4:**
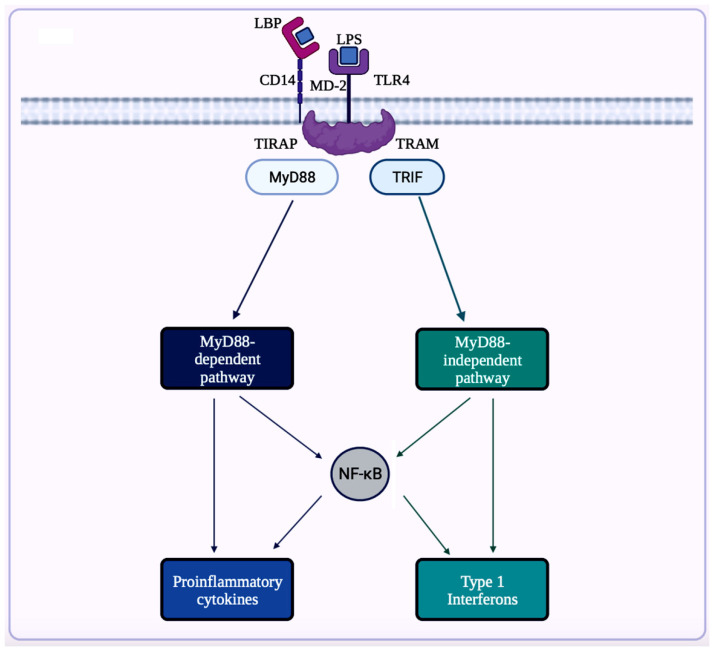
This illustration showcases an overview of LPS/TLR4 signaling. LPS can attach to LPS binding protein (LBP), which assists the transfer of LPS to glycerophosphatidylinositol-anchored protein (CD14). Then, CD14 transfers LPS to the TLR4/myeloid differentiation factor-2 (MD-2) receptor complex. After recognizing LPS, this complex triggers signals via toll-interleukin 1-receptor domain-containing adaptor protein (TIRAP) and myeloid differentiation factor 88 (MyD88) for activation of NF-κB to induce expression of pro-inflammatory cytokines. The LPS/TLR4 signaling can also be mediated via TRIF-related adaptor molecule (TRAM) and TIR domain-containing adaptor protein-inducing interferon-β (TRIF) in the MyD88-independent pathway. These adaptor molecules (TRAM and TRIF) activate the transcription factor, interferon regulatory factor-3 (IRF3), to produce type 1 interferons. This figure was created with BioRender.com.

A trigger for inflammation is the structural components of bacteria, including the by-products (e.g., SCFAs, enzymes, metabolites, LPS, cell capsule carbohydrates, and endotoxins) produced during the metabolic processes involved [64]. Chronic low-grade inflammation or inflammaging is found to cause tissue damage in most age-related diseases, including AD. One of the causes of inflammaging is dysbiosis in the gut microbiota [50]. Studies exploring the inflammation caused by an imbalance in intestinal immunity confirm the involvement of gut microbiota in innate and adaptive immunity, especially in IBD, which eventually can result in PD [32]. These studies use sterile-raised germ-free (GF) mice lacking the microorganisms existing in the gastrointestinal (GI) tract, along with mice without pathogens treated with broad-spectrum antibiotics (ABX). The ABX mice represented the innate immune system in which the myeloid cells in the bone marrow were impaired, resulting in a decrease of granulocytes and, thus, a higher likelihood of bacterial infection. In the GF mice, the development of innate lymphoid cells (ILCs) was disabled, leading to antigen receptors not being expressed. Additionally, this lack of expression affects enteric bacterial infections because the production of IL-22 decreases [32]. Considering the pathogenesis of PD is closely related to AD, the triggers for extreme inflammation could be shared between the two proteinopathies (aberrant protein aggregate diseases). Colorectal cancer (CRC) is another disease in which dysbiosis in the gut microbiota has a direct association with inflammation. A study analyzing the differences in mucosal samples between patients with CRC adenocarcinoma, tubular adenomas, and intact colon was conducted [65]. The varying bacterial diversity in mucosal samples of participants with tubular adenoma and adenocarcinoma showed signs of dysbiosis [65]. The prominent inflammatory markers generated by the gut microbiota include LPS, SCFAs, bile acids (BAs), C-reactive protein (CRP), and cytokines. The first marker mentioned, LPS, also called endotoxin, is a part of the cell wall of Gram-negative bacteria [66]. In a normal gut state, LPS (concentration ranges from 0 to 1.0 ng·mL^−1^) is prevented by the gut barrier (intestinal epithelial and mucosal layers) from entering systemic circulation and activating epithelial destruction [63,66]. However, as shown in Figure 4, LPS increased enterocyte membrane TLR-4 expression in animal models of inflammation [63].

Dysfunction of the gut barrier through numerous factors (e.g., diet, stress, pathogenic bacteria) results in gut leakiness, allowing cytokines to be activated [66]. A potential way to control inflammation is the use of SCFAs. Among SCFAs, butyrate has shown significant potential for inhibiting pro-inflammatory cytokines. In a study, nine out of thirteen patients with mild to moderate Crohn’s disease experienced a decrease in inflammation after they were administered butyrate orally [67]. This anti-inflammatory metabolite can also reverse the side effects of LPS translocation in the intestines [66]. The next biomarker related to inflammation is BAs. Due to the function of secondary BAs as strong ligands for BA receptors, the ability of anaerobic bacteria to convert primary BAs to secondary BAs is implicated in various diseases [68]. For example, in cancer, there is a higher amount of secondary BAs present [68]. Since BAs can activate inflammation through the Farnesoid X receptor (FXR) signaling pathway along with increasing the presence of *Desulfovibrionaceae*, the influence of BAs in the microbiota regarding inflammation should be further explored [69]. The next inflammatory marker is the CRP, an acute-phase reactant linked with cardiovascular disease, type 2 diabetes, and obesity [66]. In colonic inflammation, the level of the genus *Phascolarctobacterium* declines while increasing the amount of CRP. This genus also produces propionate, one of the SCFAs, which inhibits the pro-inflammatory NF-κB pathway and cytokine production [70]. Several bacterial strains, such as *Escherichia coli*, *Bacillus subtilis*, *Salmonella typhimurium*, *Salmonella enterica*, *Mycobacterium tuberculosis*, and *Staphylococcus aureus* are also capable of producing functional amyloid [71]. These functional amyloids produced by the bacteria result in the misfolding of neuronal proteins such as α-synuclein [71]. The extensive relationship between the gut microbiota and inflammation has been studied and proven to be implicated in various metabolic disorders (e.g., insulin resistance, glucose intolerance, hyperglycemia, high blood pressure, dyslipidemia, and obesity), cancer, and especially AD [66,71,72].

### 5.2. Inflammasomes in AD

Inflammasome, which is a cytosolic multiprotein complex, is implicated in causing excessive inflammation in various diseases, including autoimmune diseases, cancers, and neurodegenerative diseases [62]. In the innate immune system, if adverse stimuli (e.g., pathogens, dead cells) are detected, then inflammasomes are the receptors deployed to activate caspase-1, which contains a caspase recruitment domain (CARD), resulting in inflammation [64]. The innate immune response cascade begins with the initiation of germline-encoded pattern recognition receptors (PRRs), transmembrane or cytosolic receptors, by pathogen-associated molecular patterns (PAMPs) and damage-associated molecular patterns (DAMPs) [67]. PAMPs are the structural moieties present in microorganisms such as Gram-negative bacterial LPS, bacterial or viral nucleic acids, and bacterial peptides (e.g., flagellin). DAMPs are the endogenous molecules activated due to cellular stress, such as chromatin-associated proteins, heat-shock proteins, uric acid, and extracellular matrix fragments [67]. After the PAMPs or the DAMPs activate the cascade, PRRs such as nucleotide-binding and oligomerization domain (NOD)-like receptors (NLRs), absent in melanoma-2 (AIM-2)-like receptors (ALRs), and tripartite motif-containing (TRIM) proteins form inflammasomes.

There are three diverse types of inflammasomes: NLR-associated inflammasomes, ALR-associated inflammasomes, and the pyrin inflammasome [62]. Inflammasomes can be activated through two kinds of inflammasome signaling such as canonical and non-canonical [66]. The canonical inflammasome signaling consists of one or more inflammasome sensors, such as apoptosis-associated speck-like protein containing CARD (ASC) and caspase-1. ASC has a bipartite structure with a pyrin domain (PYD) and a CARD, both of which aid ASC in acting as an adaptor molecule. The non-canonical signaling pathway includes the activation of mouse caspase-11 or human caspase-4 and caspase-5 [64,67].

**Figure 5:**
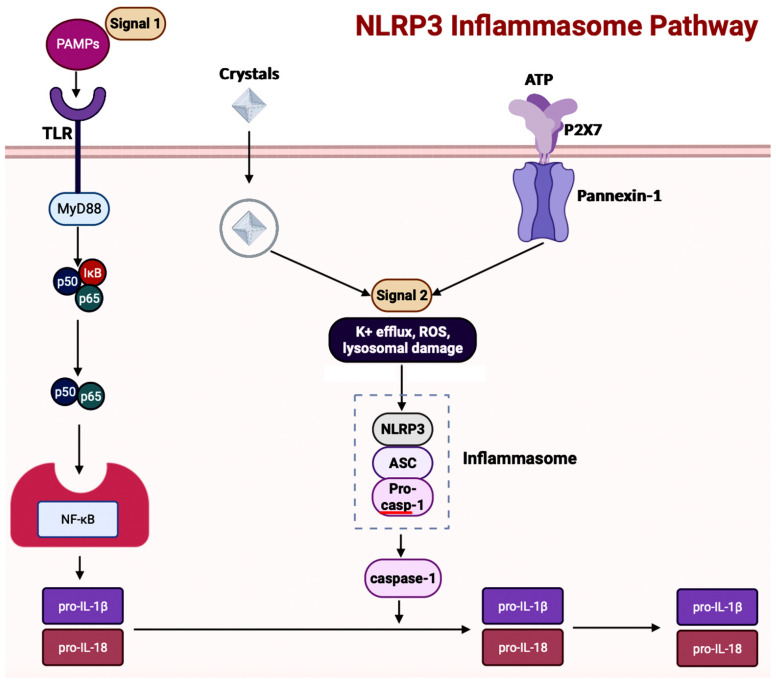
This illustration showcases the sequence of events for activation of the NLRP3 inflammasome pathway. The cascade is triggered through the binding of microbial molecules (pathogen-associated molecular patterns, PAMPSs) to the TLR receptor. NLRP3 inflammasome can also be activated through crystalline substances (uric acid and calcium pyrophosphate dihydrate). This figure was created with BioRender.com.

The NLRP3 (NOD-, leucine-rich repeat- or LRR-, and PYD-containing protein 3) inflammasome is a multimeric protein complex – which is made up of the sensor protein NLRP3, the adaptor protein ASC, and the effector protein pro-caspase-1 – having a role in development of AD [73,74,75]. When the sensor protein NLRP3 is activated, it binds to the PYD of the adaptor protein ASC, resulting in the cleavage of pro-caspase-1 into activate caspase-1 to form the NLRP3 inflammasome (Figure 5) [76]. This activates caspase-1, which then activates the inactive pro-inflammatory cytokines pro-IL-1β and pro-IL-18 into their respective mature forms [76]. Along with activating cytokines, the activated caspase-1 can also cause pyroptosis, an inflammatory-related programmed cell death [76]. Among the family of inflammasomes, NLRP3 is the most extensively studied [75].

Studies have shown the influence of the NLRP3 inflammasome in promoting neurodegenerative diseases, such as AD [73]. Specific inhibition of NLRP3 in vivo attenuated the activity of NLRP3 producing promising results, such as the decreased levels of tau and Aβ aggregates and the reduced cognitive impairment [77]. Hence, the activated NLRP3/caspase-1 inflammasome pathway can allow the accumulation of Aβ aggregates. As mentioned before, the formation of Aβ can cause a positive feedback loop in which the Aβ production is unrestricted by the microglia [77,78,79]. Apart from Aβ aggregates, inflammasomes can also influence tau pathology in AD. A study conducted in the NLRP3-inflammasome-deficient mice showed less cleaved caspase-1 and IL-1β along with a decrease in ASC formation [80]. Activation of the NLRP3 inflammasome pathway increases tau hyperphosphorylation through the production of tau kinases [80]. The implications of NLRP3 in both Aβ fibril formations and tau pathogenesis make this inflammasome a potential target for decreasing inflammation in AD.

### 5.3. Inflammasomes and GBA in AD

Inflammasome activity is influenced by alterations in the gut microbiota and diet [72]. In a ketogenic diet or calorie restriction, the NLRP3 inflammasome gets inhibited because the ketone body β-hydroxybutyrate production in the liver increases [74]. A study exploring sickness-induced anorexia analyzed the relationship between *Salmonella typhimurium* and the GBA [81]. The *S. typhimurium* effector, Slrp, was used to inhibit the inflammasome pathway to hinder anorexia [81]. In the blood and brain samples collected from patients experiencing cognitive decline, an overexpression of NLRP3 in astrocytes and microglia resulting in central inflammation was witnessed [82]. Contrastingly, the same study also analyzed the effects of dysbiosis on the activation of peripheral inflammation, consisting of the innate cells in the gastrointestinal (GI) tract. The results showed that activation of peripheral inflammasomes triggered NLRP3-mediated neuroinflammation in the brain [82]. In a study conducted, the gut microbiota from AD patients was transferred to APP/PSEN1 mice, causing microglial and NLRP3 inflammation leading to the release of inflammatory factors [83]. The GI tract then absorbs the inflammatory factors to cause inflammation. So, targeting the inflammasome signaling pathway through improving the composition of the gut microbiota would be a possible therapeutic option in AD.

### 5.4. NETosis

Neutrophil extracellular traps (NETs), produced by innate immune phagocytes or neutrophils, participate in immune regulation and pathogen clearance [84]. The structure of NETs is a large, web-like structure built with cytosolic and granule proteins and decondensed chromatin [84]. Triggers such as ROS, antibodies and immune complexes, cytokines, pathogens (e.g., bacteria, fungi, protozoa, viruses), and bacterial cell wall structural moieties (e.g., LPS) can activate the formation of NETs [84,85]. There are two pathways through which NET formation can occur: NETosis and a non-lytic form of NETosis [85].

NETosis is the primary pathway through which NETs are formed. In this pathway, the neutrophil undergoes nuclear delobulation, including disintegration of the nuclear envelope, resulting in chromatin decondensation and rupture of the plasma membrane, and most importantly, the release of NETs [85]. The non-lytic form of NETosis avoids cell death; instead, the nuclear chromatin and granule proteins are released through secreted expulsion and degranulation, respectively. Then, in the extracellular space, the components assemble into NETs [85].

One of the crucial functions of NETs is their ability to regulate inflammatory cytokines through other immune cells [85]. In atherosclerosis, microscopic cholesterol crystals trigger the release of NETs activating TLR2 and TLR4 to transcribe IL-6 and pro-IL-1β in macrophages. These activated inflammatory cytokines increase the levels of myeloid cells present in the vicinity of atherosclerotic lesions [85,86]. When studying mice lacking neutrophil proteases necessary for NETosis, the results show these mice experience lower inflammation and form smaller atherosclerotic lesions [86]. Hence, NETs have the potential to aggravate inflammation in various diseases, including AD.

Neutrophil hyperactivation is a central part of AD pathogenesis, along with neutrophil activation causing inflammation. In a study aimed to understand the influence of neutrophils in a transgenic (TG) AD mouse model, the positron emission tomography (PET) imaging showed neutrophil accumulation, increased production of cationic antimicrobial protein of molecular weight 37 kDa (CAP37), which is a neutrophil-produced molecule, and increased activation of microglia in the brain [87]. One of the most widely used animal models of AD is the penta-TG mouse model of familial AD (5xFAD) that overexpresses human APP with Swedish (K670N/M671L), Florida (I716V), and London (V717I) mutations as well as human PSEN1 with M146L and L286V mutations. Another most widely used animal model of AD is the triple TG mouse model of AD (3xTg-AD) that recapitulates both Aβ and neurofibrillary tangles (NFT) pathologies with incorporation of three mutations such as APP Swedish, microtubule-associated protein tau (MAPT) P301L, and PSEN1 M146V1 associated with FAD. A study using 5xFAD and 3xTg-AD, the widely used two TG mice models of AD, further showed evidence of neutrophil accumulation in areas with Aβ deposits [88]. The presence of neutrophil accumulation began before the onset of AD pathogenesis (microgliosis, tau phosphorylation, and cognitive deterioration). The deletion of neutrophils using an anti-Ly6G antibody significantly decreased the AD progression occurring in the model mice [88]. Neutrophil hyperactivation also has implications in causing inflammation in gut diseases such as IBD (e.g., Crohn’s disease and ulcerative colitis), CRC, and intestinal ischemia-reperfusion injury (IRI) [88].

### 5.5. NETosis and Gut Microbiota

The gut microbiota also plays a role in the case of neutrophil-led inflammation. In acute mesenteric IRI, the translocation of gut bacteria results in multiple organ failure [89]. Colonized GF mice with complex gut microbiota were analyzed to understand whether NETosis was inhibited. This study also experimented on the relationship between neutrophil-led TLR4/Toll-IL-1R domain-containing adaptor-inducing IFN-β (TRIF) signaling and NETosis. Inhibiting neutrophils decreases LPS-triggered NETosis in the colonized mice [89]. Even though NETs are important in preventing infections, overactivation of NET formation can dysregulate the intestine epithelium barrier [90]. In another study, NETs were observed to support the attachment of enteropathogenic *Escherichia coli* (*E. coli*) and Shigatoxigenic *E. coli* to the intestinal mucosa [90]. The exploration of the influence of gut flora on NET formation is important to create therapeutic options for AD.

## 6. Autophagy in AD

**Figure 6:**
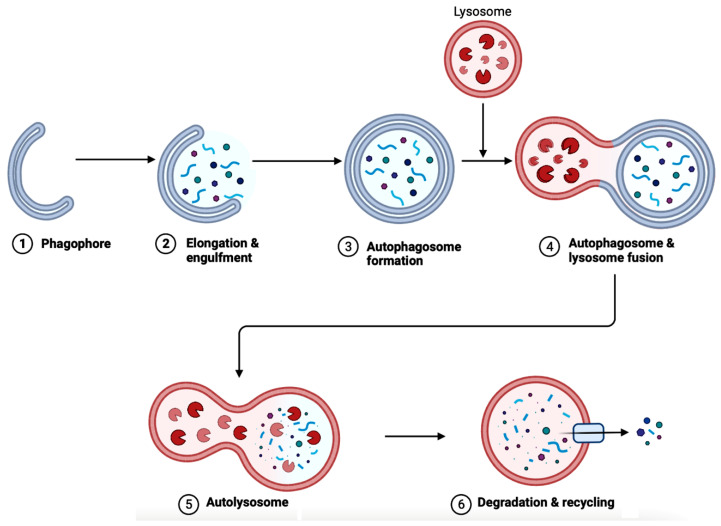
A diagram depicting the steps in autophagy. The elongation of phagophores begins when LC3 attaches to the membrane. (1) The phagophore expands when the cytoplasmic materials (e.g., mitochondria, endoplasmic reticulum, aggregated proteins, bacteria, and virus) are engulfed. (2) Then, the phagophore undergoes both elongation and engulfment. (3) This creates the structure called an autophagosome. (4) Then, the lysosome fuses with the autophagosome. (5) The fusion forms the autolysosome. (6) Lysosomal hydrolases cause degradation and recycling cellular faulty or damaged components. The process of autophagy is crucial for several functions in the body, such as cell survival, degrading protein aggregates, and homeostasis. Autophagy is impaired in AD, blocking the degradation and recycling of faulty proteins and other cellular components. This figure was created with BioRender.com.

In AD brains, the main pathological changes are the deposition of Aβ plaques and intracellular NFTs made from hyperphosphorylated tau proteins [91,92]. The accumulation of Aβ plaques begins from the dysregulation of Aβ, which is modulated by autophagy [92]. Figure 6 showcases autophagy, which is a self-degradative process that plays a critical role in maintaining cellular homeostasis [93,94]. Autophagy is required to degrade the misfolded proteins present in the brain to prevent neurodegenerative diseases. Further studies have shown that the expression of autophagy-related proteins is downregulated in AD, implying the importance of autophagy upregulation as a part of treatments for AD [95].

There are three categories of different autophagy mechanisms: microautophagy, chaperone-mediated autophagy (CMA), and macroautophagy [92]. Firstly, microautophagy involves the absorption of cytoplasmic material into a lysosome through direct invagination of the lysosomal membrane [96]. Secondly, CMA is selective in targeting certain cytosolic proteins and degrading them in the lysosomal lumen [97]. Thirdly, the unique mechanism in macroautophagy is autophagosome formation, which is used to transport the waste contents to the lysosomes [92,98].

Macroautophagy, colloquially also known as only autophagy that materializes with the participation of more than 30 autophagy-related (ATG) proteins, has been studied extensively regarding its implications in neurodegenerative diseases. Dysfunction in lysosomal degradation can result in the accumulation of autophagosomes in the dystrophic axons [98]. The ruined regulation of autophagosomes can lead to an increase in Aβ deposits because autophagosomes contain Aβ precursor protein and the necessary processing enzymes to form toxic Aβ [98]. Additionally, the exploration of mechanisms and influence of microautophagy is also on the rise. A mutation in the charged multivesicular body (MVB) protein 2B (CHMP2B), which is a part of one of the endosomal sorting complexes needed for transport (ESCRT)—explicitly ESCRT-III, is known to lead to symptoms of frontotemporal dementia and amyotrophic lateral sclerosis [96]. Another study shows overexpression of the vacuolar protein sorting 4 (VPS4), the hexameric AAA ATPase facilitates ESCRT-III filament disassembly on intracellular membranes, resulting in Aβ accumulation and MAPT phosphorylation. Apart from macroautophagy and microautophagy, CMA is also increasingly known to have an influence on neurodegenerative diseases, including AD. CMA inhibition in AD involves pathogenic variants of CMA substrates such as tau protein and the transactive response (TAR) DNA-binding protein 43 (TDP-43) [97]. The variants of tau proteins are targeted; however, the translocation of the mutants through lysosome-associated membrane protein 2A (LAMP2) is inhibited, causing the formation of irreversible oligomers at the lysosomal membrane, which suppresses CMA [97]. The mechanism for CMA inhibition varies depending on the disease explored. Therapeutic targets directed at diverse types of autophagy can be an exciting avenue for designing treatment strategies for neurodegenerative diseases, including AD.

### Implications of Gut Microbiota in Autophagy in AD

The gut microbiota has direct implications on many factors, including autophagy due to the existence of GBA. One of the ways to scrutinize whether gut flora has any effect on autophagy is to use GF mice. In the colonic epithelium of GF mice, the level of basal autophagy decreased compared to mice with intact gut flora [99]. The study also reinstated intestinal autophagy in vivo using butyrate-producing bacterial stain *Butyrivibrio fibrisolvens*. Bacteria-derived metabolites other than butyrate, such as indole-3-lactate produced by *Lacticaseibacillus*, *Lactobacillus*, *Bifidobacterium*, *Megamonas*, *Roseburia*, or *Ruminococcus* are also alternative options to induce intestinal autophagy [99]. These bacteria-derived metabolites can also modulate intestinal inflammation through the autophagy pathway. *E. coli* regulates autophagy through the NF-κB pathway, which upregulates selective microRNAs (miRNAs) before inhibiting ATG-specific proteins and then autophagy [100]. The NF-κB pathway is crucial to generating inflammatory responses; hence, maintaining the composition of the gut flora is important.

Dysfunctional autophagy and gut dysbiosis are both in a positive feedback loop due to dysregulated autophagy resulting in impaired intestinal epithelial barrier function through altering the levels of expression of the CLDN2 (Claudin-2) gene that codes for the tight junction protein Claudin-2 in the intestinal mucosa [34]. Then, the increase in bacterial translocation causes gut dysbiosis [34]. Dysregulated autophagy causes gut dysbiosis and vice versa. This imbalance in the gut flora directly impacts autophagy in intestinal epithelial cells (IECs), such as paneth and goblet cells, epididymis epithelial cells (EECs), macrophages, dendritic cells (DCs), T and B cells, natural killer (NK) cells, and nerve cells of the enteric nervous system (ENS), and CNS. Gut dysbiosis also decreases the production of antimicrobial peptides (e.g., lysozyme, α-defensin, and phospholipase A2), intestinal epithelium regeneration, degrading bacterial accumulation, and pathogenic bacteria-led immune responses.

Examining the neurons in Drosophila flies and mice, autophagy-deficient flies demonstrated a higher accumulation of ubiquitin/p62-positive protein inclusions and mitochondrial dysfunction, leading to cognitive deterioration [34]. A study analyzing in vitro and in vivo AD models along with brain tissue samples of AD patients showed sluggish autophagic flux with lower levels of degradation activity, resulting in the accumulation of autophagic vesicles [101]. Targeting gut microbiota would be realistic to decrease dysfunctional autophagy, which in turn would reduce the pathogenic features such as inflammation, oxidative stress, and accumulation of protein aggregates in many neurodegenerative diseases, including AD.

## 7. The Bioflavonoids EGCG and GS as Therapeutic Agents for AD

### 7.1. Overview of Regulating Cell Signaling by EGCG and GS

These two bioflavonoids, EGCG and GS, have shown major influences on the NF-κB, mitogen-activated protein kinase (MAPK), epidermal growth factor receptor (EGFR), insulin-like growth factor (IGF), and mechanistic target of rapamycin (mTOR) signaling pathways to provide neuroprotection in neurodegenerative diseases (Table 3). Their mechanisms of action on these signaling pathways will be described below to show their therapeutic efficacies in AD.

#### 7.1.1. NF-κB Signaling Pathway

The NF-κB is a transcription factor that regulates genes related to inflammation and innate immunity [102]. In the 3′ position of the EGCG chemical structure, there are galloyl and hydroxyl groups related to anti-inflammatory properties. In human colon cancer cells, EGCG can inhibit the DNA-binding activity of NF-κB to block the progression of the inflammatory pathway [102,103]. In a study using normal human epidermal keratinocytes, 10–40 µM of EGCG was used to halt ultraviolet B (radiation wavelength from 290–320 nm) or UVB-mediated activation of NF-κB [102]. When the cells are treated with EGCG, the anti-inflammatory mechanism begins with inhibiting TNF-α-induced NF-κB through the dissociation of the nuclear factor E2-related factor 2 (Nrf2)- Kelch-like epichlorohydrin (ECH)-associated protein 1 (Keap1) complex [94]. Through translocation to the nucleus, the dissociated Nrf2 activates the transcription of genes consisting of antioxidant response elements (AREs). Then, Keap1 removes itself from the complex before inhibiting NF-κB. Similarly, GS can also inhibit the NF-κB signaling pathway. Treating with 50 µM GS for 2 h resulted in the inhibition of TLR4 expression through microglia BV-2. TLR4 inhibition directly halts the NF-κB pathway [105]. Both EGCG and GS are potential therapeutic options to inhibit this crucial inflammatory pathway; hence, a combination therapy seems to bring in higher efficacy in inhibiting neuroinflammation in AD.

#### 7.1.2. MAPK Signaling Pathway

MAPK modules are implicated in various signal transduction pathways involved in cell proliferation, cell differentiation, and cell death [106]. There are three MAPK families: extracellular signal-regulated kinase (ERK), Jun kinase/stress-activated protein kinase (JNK/SAPK), and p38 MAPK [107]. For each of the cascades, three to five enzymes are activated in the series: a MAPK kinase kinase kinase (MAP4K), a MAPK kinase kinase (MAP3K), a MAPK kinase (MAPKK), MAPK, and MAPK-activated protein kinases [107,108]. Each of the cascades is activated through specific extracellular signals. The cascade begins with MAP3K being activated through a small GTPase and/or phosphorylation by protein kinases from cell surface receptors [106]. The MAP3K activates the MAPKK, which double-phosphorylates the MAPK. The activated MAPK then phosphorylates substrates in the cytosol and nucleus to trigger the gene expression necessary for responses [106].

The effect of EGCG on the MAPK pathway was analyzed in varying studies. A study showed that EGCG downregulated the MAPK signaling pathway by decreasing the oxidative stress in the biosynthesis of aflatoxin B1 [111]. Using the MAPK pathway, EGCG was also able to decrease cigarette smoke-stimulated inflammation in human cardiomyocytes [110]. EGCG was also involved in decreasing the expression of NLRP3 inflammasome and inflammasome-related generation of caspase-1, IL-1β, and IL-18, which also decreased the activity of the MAPK pathway [109]. In another study, EGCG downregulated the MAPK signaling pathway, resulting in decreased apoptosis and overexpression of brain-derived neurotrophic factor (BDNF) [109]. In multiple studies, due to the poor bioavailability of EGCG, results were not published; however, EGCG, in combination with another therapy, could be the way to overcome the issue of bioavailability [110].

For a potential combination therapeutic option regarding inhibiting the MAPK pathway, GS is effective. In a study exploring the expression analysis of p38 MAPK and homocysteine-induced gene (HCY-2), GS can reduce Aβ_31–35_ peptide-mediated toxicity through inhibition of p38 MAPK and HCY-2 [112]. GS has shown potential as a p38 MAPK inhibitor. The primary cultures of rat cortical neurons from the cerebral cortex showed a reduction in Aβ_31–35_ toxic peptide-induced cell toxicity after being treated with 0.5 µM of GS [112]. Inhibition of the p38 MAPK pathway can prevent neuronal cell death; hence, EGCG and GS would be an interesting combination for therapeutic exploration for inhibiting the MAPK pathway. Since multiple studies have shown the association between the MAPK pathway and AD pathogenesis, further studies focusing on increasing the bioavailability of EGCG and GS would be valuable for the field.

#### 7.1.3. EGFR Signaling Pathway

The EGFR, also known as erythroblastic B1 (ErbB1)/human epidermal growth factor receptor-1(HER-1), is a transmembrane receptor tyrosine kinase implicated in various cancers (e.g., non-small-cell lung cancer, metastatic CRC, glioblastoma, head-and-neck cancer, pancreatic cancer, and breast cancer) [113]. The structure of this receptor contains an extracellular ligand-binding domain, a transmembrane region, and a cytoplasmic tyrosine-kinase region flanked by non-catalytic regulatory regions [114]. Activated EGFR can initiate a downstream cascade affected phosphoinositide 3-kinase (PI3K), phospholipase C-gamma1 (PLC-γ1), Akt, Ras, Raf, and MAPK [115]. The ERK and JNK signaling pathways are also activated, resulting in gene expression and cell proliferation. Another pathway activated by EGFR that is important in tumors is the PI3K-dependent Akt signaling pathway [115].

Even though EGFR is extensively known and studied for its involvement in cancers, studies have shown that EGFR inhibitors are a feasible therapeutic option to reduce cognitive decline in AD [116]. In a Phase III clinical study, an anti-cancer drug (masitinib) inhibited microglia activation, controlled the Aβ and tau signaling pathway, and decreased cognitive damage [117]. The issue with using anti-cancer drugs such as gefitinib and erlotinib is the blood-brain barrier (BBB) permeability is not optimum for AD, but the EGFR inhibitors also showed neuroprotective potential in spinal cord injury [116].

Exploring an inhibitor option, EGCG can function as a tyrosine kinase inhibitor towards activated EGFR in cancer cells by regulating the phosphorylation of EGFR [118]. The inhibition of EGFR halts cell proliferation, migration, and invasion [109,119]. GS also inhibited EGFR phosphorylation and downregulated the MAPK, PI3K, Akt, and mTOR pathways in breast and prostate cancer cells [120]. Combining both EGCG and GS for a therapeutic strategy has the potential for inhibition of the EGFR signaling pathway, leading to neuroprotection in AD.

#### 7.1.4. IGF Signal Transduction Pathway

Insulin-like growth factors (IGFs) such as IGF-1 and IGF-2 participate in various biological responses such as cell differentiation, cell survival, and cell maintenance [120,121]. IGF-1 inhibits cell apoptosis while promoting cell mitosis [122]. There are two signal transduction chains of IGFs that send mitotic and metabolic signals to the nucleus of the cells: the PI3K activation pathway and the MAPK pathway [122]. 

IGF-1 has the potential to directly affect the risk of dementia in AD patients because it stimulates neurogenesis in the hippocampus, which is damaged in AD pathogenesis [123]. Results have shown IGF-1 inhibiting abnormal tau phosphorylation and Aβ deposits in cell cultures and AD transgenic mice models [123]. In a clinical study comparing the influence of IGF-1 on the brain volume of patients, higher levels of IGF-1 prevented clinical neurodegeneration [123]. In another study using picropodophyllin (PPP), an IGF-1 receptor (IGF-1R) inhibitor, treating AβPP/PSEN1 with PPP decreased microgliosis and slowed disease progression [128].

Since EGCG can function as a receptor tyrosine kinase (RTK) inhibitor, findings show that EGCG can decrease the levels of both IGF-1 and IGF-2 [124,125]. EGCG seems to alleviate atrophy-related transcription factor forkhead box protein O1 (FOXO1) while also activating a parallel signaling pathway independent of IGF-1 and insulin’s PI3K/Akt signaling axis [129]. Like EGCG, GS also inhibits IGF-1R and p-Akt pathways, resulting in cell proliferation and halting of an increase in apoptosis [126]. Both EGCG and GS are effective in targeting the IGF signaling cascade; hence, further studies using both should be increased to prevent neurodegeneration in AD.

#### 7.1.5. mTOR Pathway

The mTOR participates in cell proliferation, apoptosis, and autophagy through varying signaling pathways [127]. Hence, the mTOR signaling pathway regulates gene transcription and protein synthesis of cell proliferation and cell differentiation. The mTOR can form two distinct complexes called the mTOR complex 1 (mTORC1) and mTORC2. This pathway is significant in cancer, arthritis, insulin resistance, osteoporosis, and neurodegenerative diseases as well [127].

As a conserved protein kinase, mTOR is crucial in maintaining a balance between protein synthesis and degradation [127,130]. In postmortem studies comparing normal brains and AD brains, the mTOR activity was higher in AD brains. The 70 kDa S6 kinase (p70S6K), which can upregulate tau, and eukaryotic translation initiation factor 4E (eIF4E) are proteins regulated by mTOR, and the activity of these proteins is significantly higher in AD brains [131]. Considering the importance of the mTOR pathway in advancing AD pathogenesis, therapeutic options should be analyzed.

As an mTOR inhibitor, EGCG has shown the potential to be an ATP-competitive inhibitor [132]. Since PI3K and mTOR are part of the PI3K superfamily, both compounds have structurally similar kinase domains. Hence. EGCG can function as a dual PI3K/mTOR inhibitor [132]. EGCG can also delay apoptotic cell death through upregulating autophagy-dependent survival even with the lack of growth arrest and DNA damage-inducible protein 34 (GADD34), which is crucial in controlling apoptotic cell death via mTOR-AMPK pathways [133]. This study was also able to confirm that EGCG-induced autophagy extends cell viability in an mTOR-dependent manner [133]. In another study, GS also showed the potential to decrease phosphorylation of mTOR in endometrial cancer cells [134]. Based on the literature at present, EGCG and GS are both apt options for controlling the mTOR signaling pathway in AD.

#### 7.1.6. 5-Hydroxytryptamine Signaling

5-Hydroxytryptamine (5-HT), which is also known as serotonin, is synthesized from tryptophan [135]. This monoamine neurotransmitter is synthesized via a two-step metabolic pathway. In the first step, tryptophan is hydroxylated to 5-hydroxytryptophan (5-HTP). Then, 5-HTP undergoes decarboxylation to form 5-HT. Serotonin is produced both in the CNS by raphe neurons and in the GI tract by gut neurons and enterochromaffin cells [135]. Serotonergic dysfunction is implicated in neuropsychiatry in causing major depressive disorder.

Apart from mood disorders, research has uncovered evidence indicating the importance of serotonin regulation in other neurological disorders, such as AD. The decrease in serotonin levels in temporal and frontal lobes and cerebrospinal fluid is related to the pathogenesis of AD [136]. In the gut, serotonin is synthesized by various bacteria, such as *Corynebacterium* spp., *Streptococcus* spp., and *E. coli*. Compared to the brain, the gut has more control over serotonin synthesis due to its increased tryptophan hydroxylase 1 (TPH1) expression [136]. Another study exploring the effect of *Tuicibacter* spp. on AD pathology demonstrated decreased levels of this bacterium in AD mice [137]. Additionally, *Turicibacter* facilitates the production of serotonin in the gut. The influence of the gut is important for serotonin regulation in the body; hence, the gut flora needs to be maintained through novel methods in AD.

Both the bioflavonoids, EGCG and GS, were explored based on their respective implications on serotonin production. A rat model study, which examined the possibility of EGCG being used as an anti-depressant, showcased the increased levels of serotonin in the hippocampus after EGCG administration [138]. EGCG increased intestinal hyper-permeability and neuroprotection in the hippocampus [138]. Like anti-depressant drugs and EGCG administration, GS also increases the concentration of 5-HTP in the hippocampus [139]. In both studies, these bioflavonoids were studied using a stress model rather than AD; hence, in future studies, the influence of EGCG and GS on 5-HTP levels should be studied using the AD mice model.

### 7.2. Exploration of Bioflavonoids as Therapeutic Options in AD

Through multiple studies, EGCG and GS have been implicated in modulating the signaling pathways related to AD. However, the glaring limitation of using these selective bioflavonoids is their decreased bioavailability. Studies focused on analyzing delivery methods to optimize the absorption and usage of EGCG and GS in the body would be beneficial to develop them as novel therapeutic drugs for the treatment of AD. In this section, multiple methods with goals to administer bioflavonoids to patients are considered: AChE inhibition, diet, fecal microbiota transplantation, neural stem cell therapy, and nanomaterials.

#### 7.2.1. AChE Inhibitor

In AD, there is a change in the function of the cholinergic system. The current treatments available for AD mostly provide temporary attenuation of symptoms through cholinergic and anti-glutamatergic mechanisms [140]. Most of the currently used AChE inhibitors have side effects based on the dosage administered. For example, an overdose of Rivastigmine can result in cases of irregular heartbeat and chest pain [11]. Hence, an alternative AChE inhibitor, especially bioflavonoids, would be of interest.

In Korean red pine, the bark contains phenolics, including vanillin (VAN), catechin (CAT), and taxifolin (TAX) [141]. CAT was also shown to inhibit AChE and butyrylcholinesterase (BChE), easily allowing for reduced oxidative stress and, hence, prolonged neurotransmission. These bioflavonoids can also travel across the BBB to the nerve cells for the inhibition of both AChE and BChE to take place [141]. A study compared the five CAT compounds: CAT, EC, ECG, EGC, and EGCG. Among these compounds, only EGCG was able to inhibit both AChE and BChE with 1C_50_ values of 0.0148 µmol/mL and 0.0251 µmol/mL, respectively [142]. In other in vitro studies, neuronal cells treated with 10 µM EGCG reduced Aβ-induced cytotoxicity, with EGCG becoming an AChE inhibitor [143]. Apart from EGCG, GS can function as an efficient AChE inhibitor. In diabetic mice, GS can improve cognitive decline by inhibiting AChE [105]. The basal cholinergic neurons are affected by apolipoprotein E (ApoE) during the pathogenesis of AD. GS upregulates the peroxisome proliferator–activated receptor gamma (PPARγ), which is induced by Aβ deposits. Then, the upregulation of PPARγ results in the production of ApoE, which can decrease the deposition of Aβ [105]. EGCG and GS have the potential to function as AChE and BChE inhibitors, which can replace the current controversial AD therapeutic options on the market.

#### 7.2.2. Diet

Bioflavonoids, such as EGCG and GS, have showcased their importance in neurodegenerative diseases by regulating the microbiome and several signaling pathways. Dysbiosis, which occurs in the gut due to numerous factors and lack of diversity of the microbiota, can be improved through bioflavonoids, some of which contain antioxidant and antimicrobial properties [144]. However, the issue lies with how exactly EGCG and GS can be made available to patients. The attractive method for the availability of bioflavonoids, apart from these molecules acting as AChE inhibitors, is through diet.

EGCG is the most abundant flavonol present in green tea, and the therapeutic benefits of green tea have been attributed mostly to EGCG. The high BBB permeability of EGCG increases neuritogenesis (generation, extension, and diverging of neurites), which attenuates neurodegenerative diseases [145]. If taken along with food, the oral bioavailability of EGCG is low in humans. Studies are showing that if EGCG is taken along with nutrients such as fish oil (omega-3 fatty acids), vitamins (e.g., ascorbic acid), and minerals (e.g., selenium or chromium), then the bioavailability of EGCG improves [145]. Like EGCG, GS also deals with oxidative stress, neuroinflammation, and mitochondrial dysfunction, along with being able to cross BBB to have neuroprotective effects [146]. When the distribution of GS was analyzed, the GS concentration in the GI tract was the highest, followed by the intestine, liver, kidney, lung, heart, brain, reproductive organs, and then muscle [147]. Additionally, the GS concentration in the GI tract was enough to have anti-proliferative effects [147]. Hence, finding ways to properly administer EGCG and GS through a patient’s diet could show changes in the progression of neurodegenerative diseases, including AD.

#### 7.2.3. Fecal Microbiota Transplantation (FMT)

The GI tract is known to cause inflammatory diseases in case of gut microflora imbalance. Manipulating the microbiome through fecal microbiota transplantation is one of the most explored methods for fixing microbial imbalance [148]. The fecal matter from a healthy patient is transferred to a patient in need of regulating their microbiome [148]. The use of FMT has been prevalent in cases of *Clostridium difficile* infection (CDI) and IBD [149]. Comparing the GI tracts, microbial abundance, and diversity between IBD patients and healthy individuals showed a significant difference [150].

Among neurodegenerative diseases, FMT has also shown success in altering dysbiosis in PD patients [151]. In a transgenic mouse model treated with pre-FMT antibiotic treatment to cause dysbiosis, FMT showed the potential to decrease AD pathology [151]. However, FMT from young mice had more significant changes compared to microbiota from aged mice, which resulted in chronic low-grade inflammation or imflammaging. After the FMT in AD mice, the BBB and the metabolite levels were repaired, allowing for attenuation of AD pathogenesis [151].

Bioflavonoids can also help increase the advantages of FMT in diseases. In a study reported in 2021, FMT was conducted using microbiota from EGCG-dosed mice [152]. The results showed that microbiota retrieved from EGCG-dosed mice decreased inflammation and developed the colonic barrier integrity along with producing SCFAs and effective bacteria such as *Akkermansia* [152], also a commensal (neither harmful nor beneficial) microbe making up 1–4% of gut microbes in humans. On a similar note, microbiota from GS-dosed mice increased SCFA production and revived the gut flora, allowing the recipient mice to live longer [153]. The efficiency of FMT with EGCG and GS should be further explored for administering them as an alternative therapeutic strategy for AD patients.

#### 7.2.4. Neural Stem Cell Therapy

Neural stem cells (NSCs) are pluripotent stem cells that exist solely in the CNS. NSCs can proliferate and differentiate into multiple cell types (e.g., neurons, oligodendrocytes, and astrocytes) [154,155]. Cellular therapy makes use of neurogenic or non-neurogenic cells to improve nerve repair and tissue damage, and this method has been widely used to treat CNS diseases [155]. Neural stem cell therapy uses a mechanism of regulating the local microenvironment, increasing blood vessel development and neuron regeneration, and attenuating inflammatory responses [155]. A mice model study used NSCs obtained from the fetal brain tissue, showing the hippocampus of the recipient 3xTg-AD mice improving cognitively through enhanced endogenous synaptogenesis [156]. Further studies have shown human brain-derived NSCs (hNSCs) injected into the hippocampus of APP/PSEN1 model of AD, resulting in a development of neuronal connectivity and metabolic activity, which allowed for a decrease in AD pathogenesis [157]. Other than attenuating cognitive defects, NSCs can also inhibit inflammatory responses, neuronal loss, and regulation of microglia function [158,159].

Tea polyphenols can increase the survival rate of NSCs based on the concentration administered [160]. A study focused on the differentiation of NSCs from mouse cochlear (a fluid-filled, spiral cavity in the inner ear) in which researchers found EGCG stimulating the proliferation and neurosphere formation in the isolated NSCs in vitro [161]. In a study, ischemic stroke was induced in NPCs, and the effect of EGCG in vitro and in vivo was analyzed. After 14 days of treatment with EGCG, the neuronal differentiation was increased in the cultured NPCs [162]. Unfortunately, there are no studies with GS, specifically regarding NSCs. However, a study was conducted in which GS and daidzein (an isoflavone found exclusively in soybeans and other legumes) increased the hippocampus neuronal cell viability and proliferation in vitro [163]. Further studies should be explored to understand the influence of GS on NSC therapy for AD patients. A combination of EGCG and GS could further promote the proliferation and differentiation of NSCs in the treatment of AD.

#### 7.2.5. Nanomaterials

The major issue with administering polyphenols is their low bioavailability. The reasoning can be attributed to intrinsic factors (e.g., chemical structure, molecular weight, and low hydro solubility or solubility in water) and extrinsic factors (e.g., low stability in the GI tract, extensive Phase I and Phase II metabolism, and rapid elimination [164]. A solution to combat this low bioavailability is using polymeric nanoparticle-based delivery systems, which deliver bioactive molecules across the GI tract to target organs [164]. A nanoparticle refers to a small particle that ranges between 1 to 100 nm in size [165]. There are a variety of nanoparticle systems, such as nanospheres (NSs), nanocapsules (NCs), solid lipid nanoparticles (SLNs), cyclodextrins (CDs), liposomes (LSs), and micelles (MCs). Specifically, for polyphenols, biodegradable and biocompatible polymers are the most explored as a nanoparticle system [164].

Existing studies confirm the possibility of delivering EGCG and GS with different nanoparticles. EGCG was loaded into heat-treated β-lactoglobulin (β-Lg), which stabilizes the structure of the bioflavonoid and helps protect its antioxidant properties [166]. Further studies reaffirm the advantages of using nanomaterials to deliver EGCG. The absorption of EGCG in the GI tract can be improved by using chitosan/trimeric phosphate nanoparticles [167]. With the bioavailability of EGCG increased orally, the amount of EGCG available to plasma and jejunum also increased [167]. Shifting the focus to GS regarding clinical applications, which also have rapid metabolism and excretion, and low oral bioavailability [168]. A different approach than oral administration, this study explored options to administer GS through the nasal route. Due to the difficulty in reaching the brain through nose-to-brain delivery, chitosan (biodegradable and biocompatible natural polymer) nanoparticles are a highly feasible alternative option to increase the neuroprotective effects of GS. The mucoadhesive polymers can reside in the nasal pathway longer, regulating drug release and intracellular uptake [168]. The results regarding the efficacy of using EGCG and GS in tandem with nanoparticles look positive and highly promising for the treatment of AD; hence, this is a therapeutic drug delivery method that should be further explored in detail for clinical trials.

## 8. Conclusions and Future Directions

AD is a neurodegenerative disease that is commonly known to cause dementia among the elderly population [3]. The existing AD treatments focus more on alleviating symptoms rather than eliminating the root causes of the disease progression. A key influence on AD pathogenesis is the gut microbiota, in which dysbiosis can lead to various symptoms such as inflammation, increased BBB permeability, deposition of amyloid fibrils in the brain, and increased production of ROS [49]. Bioflavonoids are being explored as a plausible therapeutic option due to their use in diet and abundant natural obtainability and, consequently, low side effects as opposed to the synthetic and other AD therapeutic agents currently on the market.

This review article focused on the influences of EGCG and GS on different signaling pathways, gut microbiota, and therapeutic options for AD. Both EGCG and GS were implicated in influencing the NF-κB, MAPK, EGFR, IGF, mTOR, and serotonin pathways, reaffirming their potential to serve as a new benchmark for AD treatments. A feasible methodology for delivering these bioflavonoids to AD patients was also explored. One of the methods was developing EGCG and GS into AChE inhibitors to regulate the cholinergic system [105,142,143]. The next was diet, in which it was concluded that the bioavailability of both bioflavonoids would have to be increased for efficient results [145,147]. FMT suggested a better alternative regarding administering EGCG and GS, with studies proving increased efficiency of the procedure [151,152]. The next method explored was neural stem cell therapy, which highlighted the potential to heal repaired neurons. Favorably, EGCG and GS increase the proliferation and differentiation of NSCs [161,162,163]. The final plausible method for increasing bioavailability was delivering bioflavonoids through polymeric nanoparticle-based delivery systems [168]. Studies show that the bioavailability of EGCG and GS was improved using nanoparticles [168]. Studies so far strongly suggest that EGCG and GS are important bioflavonoids to target the gut microbiome to fix dysbiosis, control activation of inflammasomes to attenuate neuroinflammation, and modulate cell signaling pathways to promote autophagy in various AD models in vitro and in vivo. So far, the preclinical and clinical studies conducted on EGCG and GS have been on individual flavonoids, so combining both bioflavonoids has the potential to increase the efficiency and bioavailability of these compounds. The major features of AD include neuronal accumulation of Aβ plaques, NFTs in the temporal lobe of the brain, inactive cholinergic transmission, activation of microglia and astrocytes [169,170], all of which and a few more recently revealed features of AD (e.g., gut dysbiosis, inflammasomes, sluggish autophagy) are the potential targets of EGCG and GS for mitigation of AD pathogenesis. Overall, with further preclinical studies exploring delivery methods and efficiency of the treatments, EGCG and GS alone or in combination have the potential to become a new paradigm for the treatment of AD in clinical settings in the future.

## Figures and Tables

**Table 1:** The AChE inhibitors now available or unavailable on the market for clinical use in AD.

AChE Inhibitor	Dosage (mg/d)	Outcomes and Side Effects	Availability for Clinical Use	Reference
Tacrine	80–160	Nausea and abnormal liver functionality	No	[10]
Physostigmine	36	Nausea, diarrhea, and dizziness	No	[11]
Rivastigmine	6–12	Nausea, vomiting, and diarrhea	Yes	[11]
Galantamine *	20–50	Nausea, vomiting, and diarrhea	Yes	[11]
Donepezil	10	Higher cognitive improvements and reduced inflammatory cytokines as well as oxidative stress	Yes	[12]
	5	Reduced inflammatory cytokines and oxidative stress		
Metrifonate	NA	Bradycardia, rhinitis, abdominal pain, neuromuscular dysfunction, and respiratory failure	No	[13]

* Fewer side effects compared to other AChE inhibitor drugs; NA, not available.

**Table 2:** Animal model studies for exploring a connection between gut microbiota and AD.

AD Animal Model	Change in Gut Microbiota in AD Mice	Observed Pathological Symptoms	Reference
AD model mice (with varying ages)	Decreased microbial diversity and reduced SCFA levels	Amyloid deposition and ultrastructural abnormalities in the intestine, cognitive dysfunction, and signaling pathway alterations	[51]
APP/PSEN1 mice	Decreased microbial diversity	Cognitive dysfunction	[52]
APP_SWE_/PSIΔ_E9_ mice	Varied gut microbial composition	Increased cerebral Aβ pathology	[55]
APP/PS1 mice	Increased pro-inflammatory bacteria during aging	Autism and inflammatory-related disorders	[56]
ApoE-/- mice	*Porphyromonas gingivalis* infection	Neuronal injury	[57]

**Table 3:** Signaling pathways implicated in AD pathogenesis and use of EGCG and GS for amelioration of AD pathogenesis.

Signaling Pathway in AD	Associated Functions	EGCG and GS	References
NF-κB pathway	Regulates pro-inflammatory genes	Both can inhibit the pathway	[33,92,93,94,95]
MAPK pathway	Regulates apoptosis, differentiation, etc.	Both can inhibit the pathway	[96,99,100,101,102]
EGFR pathway	Regulates gene expression and cell proliferation	Both can inhibit the pathway	[103,104,105,106,107,108,109,110]
IGF signal transduction pathway	Regulates cell differentiation, cell survival, and cell maintenance	Both can inhibit the pathway	[110,111,112,113,114,115,116,117,118]
mTOR pathway	Regulates cell proliferation, apoptosis, and autophagy	Both can inhibit the pathway	[119,120,121,122,123]
5-Hydroxytryptamine signaling pathway	Regulates serotonin production	Both can facilitate the pathway	[124,125,126,127]
